# Hypoglycemic and hypolipidemic activity of ethanolic extract of *Salvadora oleoides* in normal and alloxan-induced diabetic rats

**DOI:** 10.4103/0253-7613.40485

**Published:** 2008

**Authors:** J.P. Yadav, Sushila Saini, A.N. Kalia, A.S. Dangi

**Affiliations:** Department of Biochemistry and Genetics, MD University, Rohtak, Haryana, India; 1 Department of Pharmaceutical Sciences, MD University, Rohtak, Haryana, India

**Keywords:** Antidiabetic, arteriosclerosis, diabetes mellitus, hypercholesterolemia, sulphonylureas

## Abstract

**Objective:**

To find out the hypoglycemic and hypolipidemic activity of an ethanolic extract of the aerial part of *Salvadora oleoides* Decne in euglycemic and alloxan-induced diabetic albino rats.

**Materials and Methods:**

Diabetes was induced in albino rats by administration of alloxan monohydrate (120 mg/kg, i.p.). Normal as well as diabetic albino rats were divided into groups (*n = 6*) receiving different treatments: vehicle (control), ethanolic extract (1 g and 2 g/kg b.w), and standard antidiabetic drug tolbutamide (0.5 g/kg b.w.). Blood samples were collected by cardiac puncture and were analyzed for blood glucose and lipid profile on days 0, 7, 14, and 21.

**Results:**

The ethanolic extract of *S oleoides* produced significant reduction (*P* < 0.001) in blood glucose and also had beneficial effects (*P* < 0.001) on the lipid profile in euglycemic as well as alloxan-induced diabetic rats at the end of the treatment period (21^st^ day). However, the reduction in the blood glucose and improvement in lipid profile was less than that achieved with the standard drug tolbutamide.

**Conclusion:**

We concluded that an ethanolic extract of *S oleoides* is effective in controlling blood glucose levels and improves lipid profile in euglycemic as well as diabetic rats.

Diabetes mellitus is heterogeneous primary disorder of carbohydrate metabolism with multiple etiological factors; it generally involves absolute or relative insulin deficiency, or insulin resistance, or both. Whatever the cause, diabetes ultimately leads to hyperglycemia, which is the landmark of this disease syndrome.[1] NIDDM has also been associated with an increased risk for premature arteriosclerosis due to increase in triglycerides and low density lipoprotein levels. About 70-80% of deaths in diabetic patients are due to vascular disease. An ideal treatment for diabetes would be a drug that not only controls the glycemic level but also prevents the development of arteriosclerosis and other complications of diabetes.[2]

Long before the use of insulin became common, indigenous remedies were used for the treatment of diabetes mellitus and hyperlipidemia. There has been an increasing demand from patients for the use of natural products with antidiabetic and antihyperlipidemic activity. This is largely because insulin cannot be used orally and insulin injections are associated with the risk of hypoglycemia and impairment of hepatic and other body functions. The undesirable side effects and contraindications of synthetic drugs, and the fact that they are not suitable for use during pregnancy, have made scientists look towards hypoglycemic agents of plant origin.[3] Many herbs and plant products have been shown to have antihyperglycemic and antihyperlipidemic action.[4–6]

*Salvadora oleoides* Decne (Salvadoraceae family), commonly known in India as *meetha jal* is an oil-yielding medicinal and multipurpose tree. It can grow in arid and alkaline conditions.[7] The leaves of *S. oleoides* are used to relieve cough and for treatment of enlarged spleen and fever. The leaves of *S. oleoides* are said to possess anti-inflammatory, analgesic, and antiulcer activity.[8] The objective of the present study was to evaluate the hypoglycemic and hypolipidemic activity of an ethanolic extract of the aerial parts (stem and leaves) of *S. oleoides* in normal and alloxan-induced diabetic rats.

## Materials and Methods

### Animals

Adult albino rats of 16-19 weeks age and of either sex, weighing 180-200 g, were procured from the Disease-Free Small-Animal House, Haryana Agricultural University, Hissar (India). The animals were kept in clean and dry plastic cages, with 12 h: 12 h light-dark cycle at 25 ± 2°C temperature and 45-55% relative humidity. The animals were fed with standard pellet diet (Hafed, Rohtak) and water was given *ad libitum.* For experimental purpose the animals were kept fasting overnight but allowed free access to water. The Institutional Animal Ethics Committee of the Department of Biosciences, M. D. University, Rohtak approved the study.

### Preparation of the extract

Aerial parts (stem and leaves) of *S. oleoides* Decne (Salvadoraceae) were collected in April-June from Matanhail, Jhajjar District, Haryana (India). The plant material was authenticated by the FRI, Dehradun. A voucher specimen (No. 14/153533) was deposited at FRI, Dehradun.

The plant material was dried under shade and powdered in a grinder. The powdered material (100 g) was extracted with 70% ethanol by hot continuous percolation method in a Soxhlet apparatus. The extract was evaporated to dryness under vacuum and dried in a vacuum desiccator to obtain a residue of 13.65 g.

### Drug administration

The quantities of the individual drugs to be administered were calculated and suspended in vehicle (1% w/v suspension of carboxymethylcellulose (CMC) in water 10 ml/kg b.w). The drug was administered continuously for 21 days orally using an infant feeding tube. The results were compared with that of the standard drug tolbutamide which was also given continuously for 21 days.

### Determination of LD_50_ of S. oleoides

LD_50_ was calculated by the probit analysis method.[9]

### Induction of experimental diabetes

A single dose (120 mg/kg b.w, i.p.) of alloxan monohydrate (1%) dissolved in sterile normal saline was used for induction of diabetes mellitus in the rats. Diabetes was confirmed 1 week after alloxan injection by determining the blood glucose concentration; only animals with blood glucose of 200-300 mg/dl (mild diabetes) were used for the experiment. The diabetic animals were allowed free access to tap water and pellet diet and were maintained at room temperature in plastic cages.

### Collection of blood and experimental setup

The rats were anesthetized with diethyl ether and blood samples were drawn from the heart of the animals. The rats were divided into two groups as follows:

1. Euglycemic rats: Euglycemic rats were divided into four groups, each group having six animals. Group I served as control and received 1% w/v suspension of CMC in water at a dose of 10 ml/kg b.w. Group II and III received ethanolic extract of *S. oleoides* in 1% CMC at a dose of 1 g and 2 g/kg b.w, respectively. Group IV received the standard drug tolbutamide at a dose of 0.5 g/kg b.w. Blood glucose and lipid profile were estimated before starting the treatment and weekly (7, 14, and 21 days) thereafter up to the end of treatment period.
2. Diabetic rats: Diabetic rats were also divided in to four groups as described above. Blood glucose and lipid profile were determined at day 0, 7, 14, and 21.

### Estimation of blood glucose and lipid profile

Fasting blood glucose was determined using the ortho-toluidine method.[10] Total cholesterol estimation was done using the Erba diagnostic kit.[11] Serum triglycerides were estimated using Enzokit (Ranbaxy).[12] HDL-cholesterol was determined using the Erba diagnostic kit.[13] VLDL (very low density lipoproteins)-cholesterol was calculated as: triglycerides/5; LDL (low density lipoproteins) cholesterol was calculated by the equation:

LDL-cholesterol = total cholesterol − (HDL + VLDL).

All estimations were done using the Erba Transasia auto-analyzer.

### Statistical analysis

The blood glucose and lipid levels before and after the administration of extract were compared using Student's ‘t’ test. The data on blood glucose level was also analyzed by one-way ANOVA. The minimum level of significance was fixed at *P* < 0.05.

### Probit analysis method

Ten rats were given *S. oleoides* ethanolic extract in doses of 0.5, 1, 2, 3, 5, 6, and 10 g/kg b.w. The rats were kept under observation for 21 days to monitor mortality. The percentage of mortality was calculated and values were transformed to probit scale [Table 1]. Calculating the value from table, the LD_50_ was found to be 29.51.

**Table 1:** Log concentration/probit mortality regression equation for rats exposed to different doses of S oleoides

*Doses of S oleoides (g/kg b.w.)*	*No. of rats exposed*	*% mortality*	*Log conc. (X)*	*Probit*	*X^2^ mortality (Y)*	*Y^2^*	*XY*
0.5	10	0	−0.3010	0	0.0906	0	0
1	10	0	0	0	0	0	0
2	10	0	0.3010	0	0.0906	0	0
3	10	0	0.4771	0	0.2276	0	0
5	10	0	0.6990	0	0.4886	0	0
6	10	30	0.7781	4.48	0.6055	20.07	3.48
10	10	40	1	4.75	1	22.56	4.75
			ΣX = 2.95	ΣY = 9.23	ΣX^2^ = 2.50	ΣY^2^ = 42.63	ΣXY = 8.27

X = 0.422 Y = 1.318

Regression equation Y = −0.15 + 3.484X

Correlation factor for SSX = ΣX^2^ − (ΣX)^2^ / N = 1.26 Correlation factor for SSY = ΣY^2^ − (ΣY^2^) / N = 41.74 Correlated sum of products of XY (C) = ΣXY − (ΣX. ΣY)/N = 4.39

In the integration equation equation Y = a + bX is the slop value; where ‘b’ = C /SSX = 3.484; and ‘a’ = Y − b.X = − 0.15; Y = a + b X: If ‘Y’ = 5; 5 = −0.15 + 3.484 X; X = 1.47;

LD_50_ was calculated by formula; LD_50_ = X log; = log 1.47 (antilog 29.51); thus, the LD_50_ was 29.51

## Results

### Effect of ethanolic extract of S oleoides on euglycemic rats

**Figure 1:**
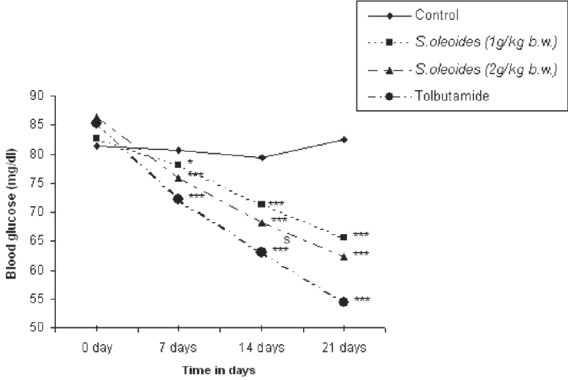
Effect of *S oleoides* on blood glucose level in euglycemic rats. Significantly different from control (*n* = 6), *P < 0.05;**P < 0.01;***P < 0.001

A significant reduction (*P* < 0.001) in blood glucose levels was observed at the end of the second week (14^th^ day) of treatment with ethanolic extract (1 g/kg and 2 g/kg b.w.) of *S. oleoides* in the euglycemic rats; this was further lowered after 21 days of treatment. The maximum reduction in blood glucose level was seen at a dose of 2 g/kg b.w, the fall being 10.98, 19.99, and 26.92%, respectively, after 7 days, 14 days, and 21 days of *S. oleoides* extract administration. However the effect of *S. oleoides* ethanolic extract was less than that of tolbutamide, which showed 36.05% reduction in blood glucose levels after 21 days of treatment [Figure 1].

As the blood glucose-lowering effect of 2 g/kg b.w. of the ethanolic extract was more, only the effect of this dose on the lipid profile of normal as well diabetic albino rats is shown in Table 2. Administration of the ethanolic extract led to a significant fall (*P* < 0.001) in the level of triglycerides, total cholesterol, LDL, and VLDL, and improved the HDL levels, in normal rats. Tolbutamide also showed reduction in the levels of triglycerides, total cholesterol, LDL, and VLDL, and improved the HDL, after 21 days [Table 2].

**Table 2:** Effect of ethanolic extract (2 g/kg b.w.) of *S oleoides* on lipid profile in euglycemic rats

Lipid profile	*Period*	*Control*	*Ethanolic extract*	*Tolbutamide*
TG	0 day	84.46 ± 1.50	82.28 ± 1.65	85.32 ± 1.78
	7 days	80.32 ± 2.08	77.25 ± 1.82***	76.21 ± 1.63***
	14 days	81.04 ± 1.70	71.02 ± 1.46***	69.35 ± 1.14***
	21 days	80.61 ± 1.94	64.70 ± 2.01***	62.03 ± 1.46***
TCH	0 day	78.23 ± 1.75	79.06 ± 1.89	76.14 ± 1.23
	7 days	79.13 ± 0.71	73.72 ± 1.06***	70.09 ± 1.37***
	14 days	77.65 ± 1.30	69.08 ± 1.57***	65.43 ± 1.11***
	21 days	76.45 ± 0.90	62.56 ± 1.78***	58.22 ± 1.93
HDL	0 day	22.18 ± 1.01	18.21 ± 1.19	21.65 ± 1.08
	7 days	21.06 ± 1.82	19.42 ± 1.42^NS^	22.78 ± 0.98^NS^
	14 days	22.47 ± 1.13	20.22 ± 1.53*	24.61 ± 1.02***
	21 days	39.16 ± 1.32	21.52 ± 1.23***	26.52 ± 1.36***
LDL	0 day	42.01 ± 1.04	44.40 ± 2.16	42.67 ± 1.72
	7 days	38.98 ± 1.77	38.85 ± 1.89***	34.25 ± 1.53***
	14 days	39.41 ± 1.53	34.66 ± 2.31***	29.72 ± 1.19***
	21 days	16.98 ± 1.50	28.70 ± 1.87***	25.92 ± 1.02***
VLDL	0 day	16.89 ± 1.50	16.45 ± 1.42	17.81 ± 1.24
	7 days	16.06 ± 1.81	15.45 ± 0.92^NS^	15.87 ± 1.17^NS^
	14 days	16.20 ± 1.62	14.20 ± 1.40*	13.93 ± 1.34*
	21 days	16.12 ± 1.60	12.94 ± 0.74***	12.36 ± 1.35***

Values are mean ± SD, *n* = 6 in each group. NS = nonsignificant

* *P* < 0.05

**P < 0.01

*** *P* < 0.001 when compared with the vehicle-treated group

### Effect of S oleoides ethanolic extract on alloxan-induced diabetic rats

**Figure 2:**
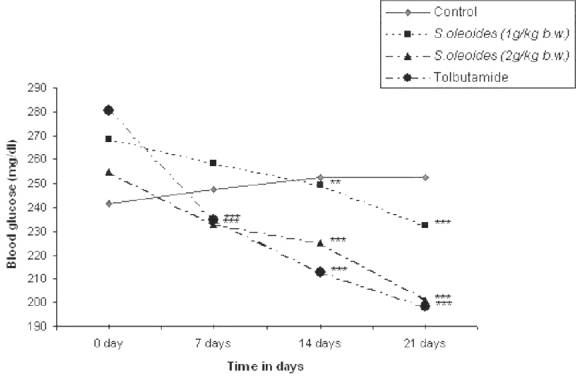
Effect of *S oleoides* on blood glucose level in alloxan induced diabetic rats. Significantly different from control (*n* = 6), *P < 0.05; **P < 0.01; ***P < 0.001

On repeated administration of ethanolic extract at doses of 1g and 2 g/kg b.w.for 21 days, a significant (*P* < 0.001) dose-dependent decrease in blood glucose of the diabetic rats was seen as compared to the vehicle-treated group. Tolbutamide showed a 29.37% decrease as compared to the control group [Figure 2].

Administration of vehicle to alloxan-induced diabetic rats resulted in an increase in the level of triglycerides, total cholesterol, LDL, and VLDL, and decreased HDL, after 21 days. Continuous administration of the ethanolic extract (2 g/kg b.w.) of *S. oleoides* led to significant decrease (*P* < 0.001) in the level of triglycerides, total cholesterol, LDL, and VLDL in the diabetic rats, while it increased (*P* < 0.01) the level of HDL [Table 3].

**Table 3:** Effect of ethanolic extract (2 g/kg b.w.) of *S oleoides* on lipid profile in alloxan-induced diabetic rats

Lipid profile	*Period*	*Control*	*Ethanolic extract*	*Tolbutamide*
TG	0 day	130.06 ± 3.42	122.05 ± 3.31	132.26 ± 3.04
	7 days	132.27 ± 3.76	119.46 ± 3.62***	127.34 ± 3.41***
	14 days	136.28 ± 3.33	108.23 ± 2.43***	116.69 ± 3.56***
	21 days	138.94 ± 2.42	99.12 ± 2.78***	101.63 ± 3.52***
TCH	0 day	125.452 ± 3.93	112.62 ± 2.76	120.11 ± 2.71
	7 days	126.34 ± 2.21	106.24 ± 2.89**	110.07 ± 2.93***
	14 days	128.60 ± 3.12	98.92 ± 1.86***	101.27 ± 2.42***
	21 days	131.45 ± 2.90	90.46 ± 1.80***	93.08 ± 2.32***
HDL	0 day	17.94 ± 1.44	15.62 ± 1.89	16.43 ± 1.87
	7 days	17.02 ± 1.63	16.95 ± 1.62^NS^	18.23 ± 1.56^NS^
	14 days	16.17 ± 1.02	17.81 ± 1.06*	19.86 ± 1.72**
	21 days	15.36 ± 1.57	18.76 ± 0.81***	21.16 ± 1.22***
LDL	0 day	81.50 ± 2.24	72.59 ± 2.15	82.51 ± 1.96
	7 days	82.85 ± 2.77	65.40 ± 2.16***	74.21 ± 2.72***
	14 days	85.18 ± 2.01	59.40 ± 2.49***	65.15 ± 2.36***
	21 days	88.04 ± 2.81	51.88 ± 2.23***	56.48 ± 2.17***
VLDL	0 day	26.01 ± 2.81	24.41 ± 1.76	27.19 ± 1.76
	7 days	26.47 ± 2.88	23.89 ± 0.72^NS^	25.98 ± 1.10^NS^
	14 days	27.25 ± 1.82	21.64 ± 1.12**	23.18 ± 1.16***
	21 days	27.79 ± 2.41	19.82 ± 0.71***	20.32 ± 1.28***

Values are mean ± SD, *n* = 6 in each group. NS = nonsignificant

* *P* < 0.05

** *P* < 0.01

*** *P* < 0.001 when compared with the vehicle treated group

## Discussion

Diabetes mellitus is a chronic disorder caused by partial or complete insulin deficiency, which produces inadequate glucose control and leads to acute and chronic complications. Premature and extensive arteriosclerosis involving renal, peripheral, and cardiovascular vessels remain the major complication of diabetes mellitus. Alteration in the serum lipid profile is known to occur in diabetes and this is likely to increase the risk for coronary heart disease. A reduction in serum lipids, particularly of the LDL and VLDL fraction and triglycerides, should be considered as being beneficial for the long-term prognosis of these patients.[14] Lowering of blood glucose and plasma lipid levels through dietary modification and drug therapy seems to be associated with a decrease in the risk of vascular disease.

In the present study, treatment with *S. oleoides* ethanolic extract (2 g/kg b.w.) in euglycemic rats produced significant decrease in blood glucose level. The hypoglycemic effect may be due to increased secretion of insulin from the b-cells of the pancreas, i.e., pancreatotrophic action.[15] *S. oleoides* contain several organic sulfur compounds and it is well known that sulfur derivatives show hypoglycemic effects. In fact, many plants containing sulfur are used traditionally as antidiabetics.[16–17] These compounds produce an increase in insulin, probably by inhibition of some substances competing with insulin for their SH-group. The results were comparable with that of tolbutamide, which acts by stimulation of insulin release,[18] thus further confirming that the extract lowers the blood glucose by a pancreatotrophic action.

Moreover, *S. oleoides* produced significant beneficial effects in the lipid profile in euglycemic rats, reducing triglycerides, total cholesterol, LDL, and VLDL, and increasing HDL, significantly. The ethanolic extract increased secretion of insulin from b-cells of pancreas; this increased secretion of insulin stimulates fatty acid biosynthesis and also the incorporation of fatty acids into triglycerides in the liver and adipose tissue.[19]

Alloxan, a beta cytotoxin, induces ‘chemical diabetes’ in a wide variety of animal species by damaging the insulin-secreting cells of the pancreas.[19] Literature sources indicate that alloxan rats are hyperglycemic.[20] The use of lower doses of alloxan (120 mg/kg b.w.) produced a partial destruction of pancreatic b-cells even though the animals became permanently diabetic.[21] Thus, these animals have surviving b-cells and regeneration is possible.[22] It is well known that the sulfonylureas (tolbutamide) act by directly stimulating the b-cells of the islets of Langerhans to release more insulin and these compounds are active in mild alloxan-induced diabetes where as they.[23] Since our results show that tolbutamide reduced the blood glucose levels in the diabetic animals, the state of diabetes is not severe.

Prolonged administration of an ethanolic extract of *S. oleoides* leads to significant reduction in blood glucose level, which is in agreement with other studies.[24,25] The hypoglycemic activity of the drug was due to the regeneration of pancreatic cells that were partially destroyed by alloxan, and potentiation of insulin secretion from surviving b-cells of the islets of Langerhans.[26]

Diabetic rats were observed to have increased plasma lipids, which are responsible for several cardiovascular disorders.[27] The higher lipid levels seen in diabetic rats was due to increased mobilization of free fatty acids from peripheral depots and also due to lipolysis caused by hormones.[28,29] The ethanolic extract leads to regeneration of the b-cells of the pancreas and potentiation of insulin secretion from surviving b-cells; the increase in insulin secretion and the consequent decrease in blood glucose level may lead to inhibition of lipid peroxidation and control of lipolytic hormones. In this context, a number of other plants have also been reported to have antihyperglycemic, antihyperlipidemic, and insulin stimulatory effects.[30–32]

It is well known that LDL plays an important role in arteriosclerosis and that hypercholesterolemia is associated with a defect relating to the lack of LDL receptors. The decrease of cholesterol and LDL levels achieved by administration of ethanolic extract, demonstrates a possible protection against hypercholesterolemia and the harm this condition brings about. Further studies are needed to identify the chemical constituents of the ethanolic extract of *S. oleoides* that may be responsible for the hypoglycemic and hypolipidemic activity.
